# Commensal *Neisseria* and Antimicrobial-Resistant Gonorrhea

**DOI:** 10.1146/annurev-micro-022024-024306

**Published:** 2025-10

**Authors:** Crista B. Wadsworth, Maira Goytia, William M. Shafer

**Affiliations:** 1 Thomas H. Gosnell School of Life Sciences, Rochester Institute of Technology, Rochester, New York, USA; 2 Department of Biology, College of Computer, Mathematical, and Natural Sciences, University of Maryland, College Park, Maryland, USA; 3 Department of Microbiology and Immunology and Emory Antibiotic Resistance Center, Emory University School of Medicine, Atlanta, Georgia, USA; 4 Laboratories of Microbial Pathogenesis, Veterans Affairs Medical Research Service, Veterans Affairs Medical Center, Decatur, Georgia, USA

**Keywords:** *Neisseria*, antibiotic resistance, microbiome, commensal bacteria, horizontal gene transfer

## Abstract

Alongside the crisis of antimicrobial-resistant gonorrhea is the threat of bystander selection on commensal *Neisseria*. As *Neisseria* species are permissive to gene flow across lineages, their evolutionary fates are irrevocably intertwined. Horizontal gene transfer (HGT) within the genus occurs through transformation and exchange of plasmids through conjugation. Both mechanisms of HGT threaten the long-term efficacy of antimicrobial treatments, with resistance passed between commensals and pathogens multiple times (e.g., mosaic *penA* and *mtr* alleles). Here, we underscore the importance of commensal *Neisseria* as a bubbling cauldron of adaptive solutions for pathogenic *Neisseria*, review the mechanisms of resistance harbored by commensals and transferred to the gonococcus, and discuss the impact of contemporary selective pressures on the future evolutionary trajectory of the genus. Ultimately, we believe that predicting the future efficacy of antimicrobials for the treatment of gonorrhea will only be successful if the commensal *Neisseria* are also considered.

## INTRODUCTION

*Neisseria gonorrhoeae*, the bacterium responsible for the sexually transmitted infection gonorrhea, has evolved antimicrobial resistance (AMR) to all antibiotics introduced for treatment since the 1930s (157). Circulating AMR compromises the management and control of gonococcal infections and provokes concern regarding untreatable gonorrhea and increasing preantibiotic-era disease complications, such as pelvic inflammatory disease, which can result in infertility, ectopic pregnancy, and chronic pelvic pain; disseminated infections, which possibly lead to arthritis, endocarditis, and meningitis; and ophthalmia neonatorum, which leads to blindness in newborns infected during vaginal birth from infected mothers (159). Contemporaneously with increasing AMR, the incidence of gonococcal infections has risen. For example, the World Health Organization (WHO) reported a 10% increase from 2012 to 2016 in cases of gonorrhea worldwide (from 78 million to 87 million), and the Centers for Disease Control and Prevention (CDC) estimated a 96% increase in infection rates from 2009 (which had the lowest reported infection rate since 1950) to 2022 in the United States (167). With no current vaccine and limited antimicrobials in development [i.e., zoliflodacin (18) and gepotidacin (147)], there is an urgent need to address the AMR crisis in gonococci to stem this wide-scale spread of gonococcal disease.

*N. gonorrhoeae* bacteria, or gonococci, are gram-negative and oxidase- and catalase-positive diplococci, and they are strict human pathogens. Though often considered colonizers of the urogenital tract and rectum, these bacteria also colonize the human pharynx, an ecological niche inhabited by closely related commensal *Neisseria* species (41). Thus, a common ancestor of all human-associated *Neisseria* likely colonized the oral cavity of an early human or humanoid (165). Modern gonococci likely only emerged after their introduction to the genital tract and the evolution of traits facilitating the polymorphonuclear leukocyte (PMN) inflammatory response and evasion of PMN-killing mechanisms; these traits aided sexual transmission (114). As to when this niche shift occurred, there are alternative hypotheses. Passages from the Old Testament, such as “When any man has a bodily discharge, the discharge is unclean,” and the Greek physician Galen’s description of a gonorrhea-like disease “as flow of seed” in the second century support the idea that gonorrhea is an ancient disease in *Homo sapiens* (156). However, limited genetic diversity in gonococcal populations suggests more recent evolutionary origins, perhaps even as recent as the 1500s (59). *Neisseria* likely have been introduced to the urogenital tract throughout human history, with more pathogen-like lineages causing symptomatic gonorrhea, some lineages persisting and others disappearing, and a well-adapted urogenital tract–residing clone expanding in recent history. Indeed, repeated sojourns to the urogenital tract have been documented for several contemporary *Neisseria* species typically considered pharyngeal area–inhabiting commensals (110, 153).

AMR to all recommended antibiotics used to treat gonorrhea has arisen rapidly since the introduction of sulfonamides in 1936 (73): After only 8 years of sulfa drug usage, resistance became widespread (44). Similarly, penicillin (introduced in 1943) was originally highly effective for the treatment of gonorrhea. However, over the next 10–15 years, penicillin minimum inhibitory concentrations (MICs) increased because of several chromosomal mutations, with high-level resistance emerging after the acquisition of a β-lactamase-encoding plasmid in the late 1970s/early 1980s (48, 68) (Table 1). Similar trends emerged for other drugs, including aminoglycosides [introduced in 1961; resistance reported in 1967 (141)], the macrolides erythromycin [1952; 1982 (108)] and azithromycin [1983; 1993 (38)], tetracycline [1962; 1985 (82, 98)], the fluoroquinolone ciprofloxacin [1987; 1990 (64)], and the extended-spectrum cephalosporins (ESCs) cefixime [1983; 2000 (146)] and ceftriaxone [1980; 2009 (106)]. Importantly, though, resistance is often not a binary phenotype: It is an accumulation of mutations, each of which reduces susceptibility by some amount alone but in combination can push MICs above a clinical breakpoint, after which clinical failures are expected. So, in conjunction with increasing numbers of isolates that have MICs above resistance breakpoints, an additional concern is clinical treatment doses increasing over time. For example, the recommended dose of penicillin doubled in 1965 (43), and the recommended dose of ceftriaxone has steadily increased since the 1990s—from 125 mg to 250 mg to 500 mg and to 1 g recently in the United Kingdom and Europe (155, 168). Above-breakpoint-resistance carriage in gonococcal populations remains high to most antibiotics (15)—for example, the 2020 Gonococcal Isolate Surveillance Project report estimates that 15% of isolates in the United States are resistant to penicillin, 20% to tetracycline, 33.2% to ciprofloxacin, and 5.8% to azithromycin—and while resistance to ESCs is lower (< 0.5%), global spread of the ceftriaxone-resistant lineage FC428 threatens the long-term efficacy of ceftriaxone monotherapy, our last recourse for first-line empiric antibiotic therapy for gonorrhea (29, 102, 116). The CDC estimates that only 44.5% of isolates remain susceptible to all antibiotics (167). Though many of the mutations imparting resistance to different classes of antibiotics have arisen de novo, increasing evidence indicates that gonococci have repeatedly acquired mutations contributing to reduced susceptibility from closely related commensal species (Table 1) (49, 67, 113, 124, 130).

Adaptive genetic variation, encoding traits such as resistance and colonization site niche shifts, flows relatively freely between *Neisseria* species as they constitutively express their competence systems and are competent for transformation through all stages of growth. This DNA promiscuity results in a highly recombinogenic consortium of species interconnected by frequent horizontal gene transfer (HGT) events, with fuzzy boundaries (10, 66, 95, 109). An estimated 6% of genes across the gonococcal genome show some evidence of gene sharing, with alleles imported from other members of the *Neisseria* genus (10, 62, 63, 91). Hence, attention on commensals as reservoirs of resistance for gonococci is increasing, with the spread of ceftriaxone and azithromycin resistance in recent years driven by recently acquired commensal alleles at the *penA* (17, 138) and *mtrR-CDE* (129, 163) loci, respectively. Therefore, in this review, we highlight the intertwined relationships between the pathogenic and commensal *Neisseria* and the resistance alleles shared across species’ boundaries, and we discuss the implications of current empiric and prophylactic therapies for future gonococcal evolutionary trajectories.

## HOUSEHOLD DYNAMICS: RELATIONSHIPS AMONG CLOSE RELATIVES AND THEIR HOST

**Figure 1:**
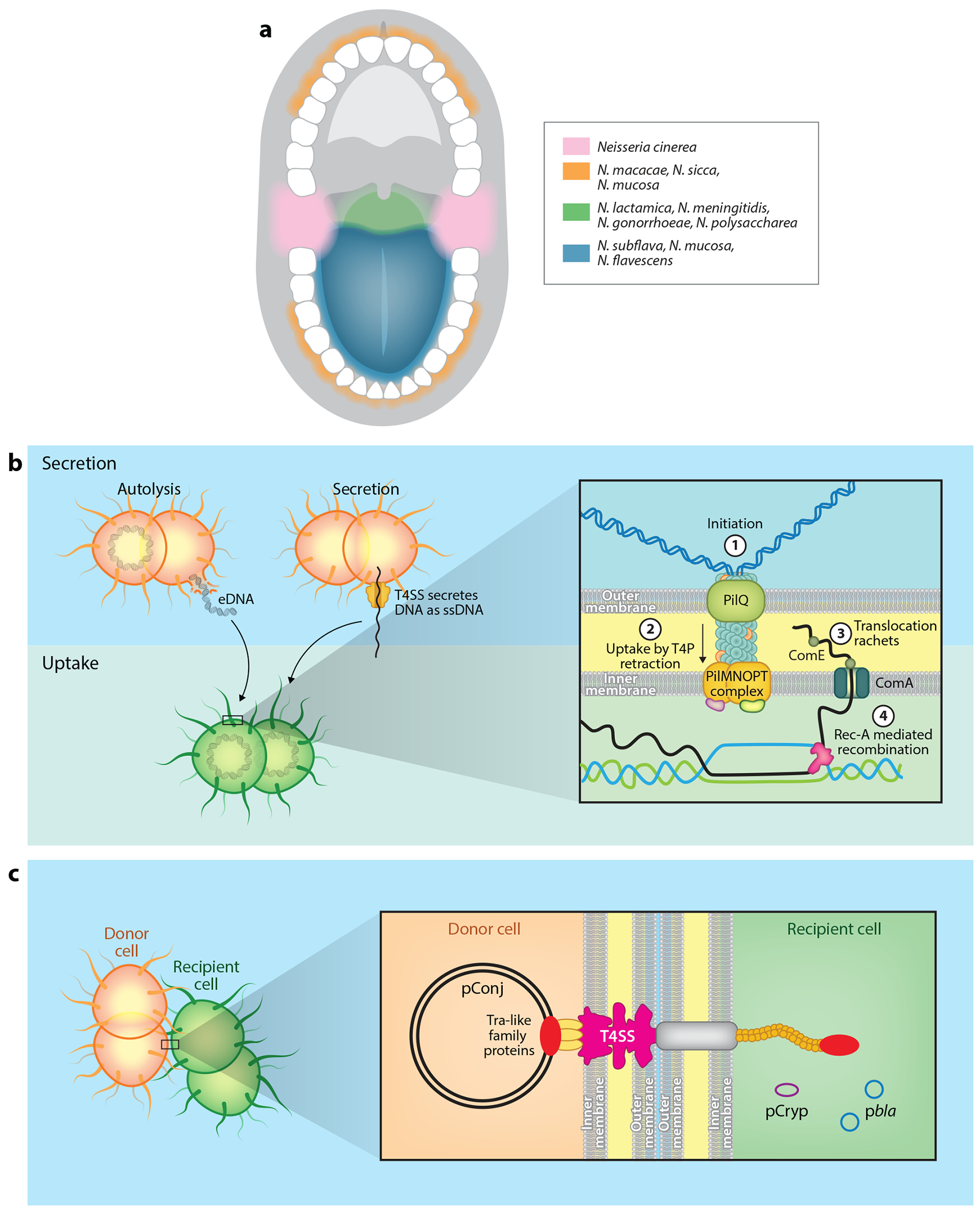
Multiple intrinsic features of neisserial biology facilitate horizontal gene transfer (HGT) of adaptive genetic variation, including antibiotic resistance. The genus *Neisseria* includes several closely related species that reside in the oral cavity of humans. (*a*) Species colonize distinct niches within the oral cavity; however, their proximity to one another facilitates HGT. (*b*) Cells are naturally competent for transformation of extracellular DNA (eDNA) from both lysed cells and secreted single-stranded DNA (ssDNA) from type IV secretion systems (T4SS) (23). Uptake of this exogenous DNA is mediated by Type IV pili (T4P), and several accessory proteins are involved in ① binding (e.g., ComP, indicated by *orange circles*, among the majoritarian PilE protein, indicated by *blue circles*, which together form the pilus), ② pilus retraction (including PilB and PilT), and ③ transport into the cell (ComE binding DNA and the inner membrane pore ComA facilitating DNA entry). Finally, ④ homologous recombination integrates DNA into the genome via RecA-mediated pathways. (*c*) *Neisseria* also share mobile genetic elements through conjugation via T4SS, with the plasmid pConj harboring the necessary machinery for conjugal transfer (i.e. the *tra*-like genes harboring the genes encoding T4P and T4SS). *Neisseria* plasmids pConj and p*bla* have been shared across species’ boundaries and impart tetracycline resistance and β-lactam resistance, respectively. pCryp’s function has yet to be assessed.

The genus *Neisseria* (family *Neisseriaceae*) contains 10 typically diplococcoid, oxidase-positive, and often catalase-positive human-associated species: *N. gonorrhoeae* (the gonococcus), *Neisseria meningitidis* (the meningococcus), *Neisseria cinerea*, *Neisseria polysaccharea*, *Neisseria lactamica*, *Neisseria mucosa*, *Neisseria oralis*, *Neisseria subflava*, *Neisseria elongata* (atypical rod), and *Neisseria bacilliformis* (atypical rod) (89). Species display a range of pathogenicity. However, most members of the genus are considered nonpathogenic members of the human pharyngeal microbiome (Figure 1a) (80, 89), and they make up the most abundant genera within *Proteobacteria* in oral and pharyngeal samples (approximately 10% of operational taxonomic units) (21, 77). While commensal species are universally carried by healthy human adults (42, 79, 160), the species that cause symptomatic disease at appreciable rates, *N. gonorrhoeae* and *N. meningitidis*, are carried at much lower frequencies (between 0.01% and 10%) (79, 161). Little is known about primary succession of the pharyngeal niche with *Neisseria*; however, *N. lactamica* clearly is an early colonizer of infants, with carriage rates spiking from 2% in children 2 weeks of age to 44% in children 56 weeks of age (16). Colonization with *N. lactamica* decreases with age (47, 88), and presumably, other commensals are introduced over time, likely from familial contact (3); however, the temporal dynamics of other *Neisseria* are poorly characterized. Interestingly, carriage of *N. lactamica* is associated with a high titer of antibodies against *N. meningitidis*, and therefore, childhood colonization with *N. lactamica* has been proposed to provide limited protection against *N. meningitidis* colonization later in life (88, 170).

Of the human-associated *Neisseria*, only *N. gonorrhoeae* and *N. meningitidis* have significant pathogenic potential. *N. meningitidis* is typically an asymptomatic colonizer that inhabits the nasopharynx and is carried in 10% of healthy adolescents and young adults, although varied rates are observed in different populations (31). Symptomatic meningococcal disease only occurs when the organism moves from its native niche into the blood or brain to cause invasive disease (meningococcemia and bacterial meningitis), which can be easily diagnosed via PMNs in the cerebrospinal fluid (32). Unlike *N. meningitidis*, which naturally resides in the nasopharynx, *N. gonorrhoeae* routinely colonizes the rectum, cervix, and urethra as well. Symptomatic gonococcal infection is characterized by a purulent discharge of gonococcal cells and PMNs, with male urethral exudate the most obvious presentation of the disease, while colonization at other sites is more frequently asymptotic and more difficult to diagnose. Despite their unique disease and carriage presentations and the presence of distinct virulence factors, the two pathogenic *Neisseria* are most alike—based on DNA sequence similarity and similar phenotypic traits—compared with the other commensal species; these parallels support the theory that both pathogens evolved from a common pharyngeal-inhabiting progenitor (132).

Though factors influencing the transition from commensal inhabitant to invasive pathogen are not well-understood, there are pathogenesis-related factors in *N. gonorrhoeae* and *N. meningitidis* typically absent in commensal *Neisseria*. Common pathogenesis-related factors between *N. gonorrhoeae* and *N. meningitidis* include IgA proteases, which cleave soluble IgA and lysosomal-associated membrane protein 1 (94); Opa adhesion proteins, which mediate host cell attachment and invasion (94); Maf polymorphic toxin/immunity systems that mediate interbacterial competition (70); and TdfF iron uptake systems required for intracellular survival (94). Additionally, key virulence factors expressed in pathogenic *Neisseria* are phase-variable (e.g., Opa and pilin), with the DNA polymerase “slipping” during replication of poly-G, poly-A, or short tandem repeats and turning gene expression on or off by altering the reading frame (128). This action introduces phenotypic diversity into the bacterial population (particularly in the presentation of cell surface proteins) and makes an effective response against the pathogen more difficult for the host’s immune system to mount. A complete list of pathogenesis-related factors can be found in other reviews (117), but defining unique genes involved in pathogenesis may be key to the development of better diagnostics, vaccine target identification, and possible therapeutics.

Type IV pili (T4P) promote colonization and adherence to cells and enable intragenus HGT through transformation (22) (Figure 1b). In *N. gonorrhoeae*, piliated strains exhibit transformation frequencies in excess of 1%, while nonpiliated strains have frequencies closer to 1 × 10^−7^ (136). T4P are formed by pilin subunits (PilE) extruded from the outer membrane through the PilQ pore complex (103, 134, 143). Extracellular DNA binding is mediated by the pilus-associated ComP protein (Figure 1b, step 1), which has a high affinity for *Neisseria*-specific DNA, through the recognition of DNA uptake sequences (DUSs). Once ComP is bound, retraction of the pilus transports ComP-bound DNA through the outer membrane and peptidoglycan layer, and the cytoplasmic NTPase motors PilF (also known as PilB), PilT, and PilU mediate this transit (53) (Figure 1b, step 2). In the periplasm, single-stranded DNA bound by ComE is imported through the inner membrane and into the cytoplasm through a pore formed by ComA (Figure 1b, step 3). Homologous recombination integrates DNA into the genome via RecA-mediated pathways (RecBCD and RecF). This intrinsic natural machinery for host colonization, DNA donation, DNA uptake, and homologous recombination is directly responsible for the extensive allelic exchange observed across the *Neisseria* genus.

Though HGT in *Neisseria* has aided in rapid, beneficial adaptive evolution, the propensity for DNA sharing may also present a weakness by introducing divergent alleles that could be deleterious in alternative genomic backgrounds (e.g., by resulting in gene disruption or maladapted gene combinations). Thus, barriers to gene exchange that limit transformation between evolutionarily distant relatives have evolved. For example, coevolution between the ComP protein and DUSs—12-bp sequences (e.g., AT-DUS: 5′-AT-GCCGTCTGAA-3′) located approximately every 1,100 bp throughout *Neisseria* genomes—has produced eight *Neisseria* clades that have a distinct ComP–DUS “dialect,” with species sharing the same dialects more frequently exchanging DNA (94). Interestingly, species sharing ComP–DUS dialects also share common colonization sites in the oropharynx and nasopharynx, and this observation provides strong evidence that DNA dialects are directly involved in reproductive isolation and speciation between *Neisseria* species (52). Another barrier to gene exchange that has emerged is divergence in the number and identity of methyltransferases between *Neisseria* species, referred to as “tribal warfare” (135). For example, methylated DNA from *N. elongata* and other commensal *Neisseria* species has been shown to kill *N. gonorrhoeae* and *N. meningitidis*. Divergence in methyltransferase activity across species (*N. gonorrhoeae* have 14 to 19 DNA methyltransferases, while the commensal *N. elongata* have only 7 to 10) differentially modifies CpG and GpC sequence motifs, produces species-specific methylation signatures, and results in restriction enzyme cleavage and the formation of double-strand breaks of chimeric DNA with foreign methylation patterns (81).

Despite barriers to HGT, DNA sharing is prevalent across *Neisseria*. Thus, the evolution of gonococci, as well as AMR in gonococci, is intertwined with that of close commensal relatives within the genus. Cross-species genetic exchange likely occurs in the oropharynx and nasopharynx, where most *Neisseria* species reside as members of the core oropharyngeal flora (85), though repeated sojourns of pharyngeal-inhabiting *Neisseria* to the urogenital tract have been reported (14, 110, 122), and thus, we cannot exclude the possibility that intragenus gene exchange occurred in these other colonization sites. Regardless of the location of HGT, *Neisseria* share DNA through transformation [wherein T4P bind to exogenous DNA and promote uptake into the cell (1), and RecA facilitates recombination of homologous sequences into the chromosome (84)] and conjugation (wherein strains harboring a conjugative plasmid, pConj, can pass both this plasmid and others within and between species) (171) (Figure 1b, step 4). Examples of cross-species chromosomal allelic exchange have been reported at multiple loci, with resultant hybrid alleles in nonnative *Neisseria* genomes referred to as mosaics. Plasmid sharing across species has been documented (171) and may become an important mechanism of resistance acquisition given newly implemented doxycycline prophylactic therapy and selection for *Neisseria* carrying pConj, which imparts resistance to tetracycline class antibiotics through *tetM* (see the section titled Rapid Shuttles for Genetic Exchange: Plasmids as Alternative Drivers of Resistance Acquisition). However, further characterization of plasmids across all *Neisseria* is needed to understand the overall significance of these mobile genetic elements and the future evolution of AMR in *Neisseria*.

## SHARED FAMILY ARTIFACTS: COMMENSAL ALLELES TRANSFERRED TO PATHOGENS

### Penicillin-Binding Proteins

**Figure 2:**
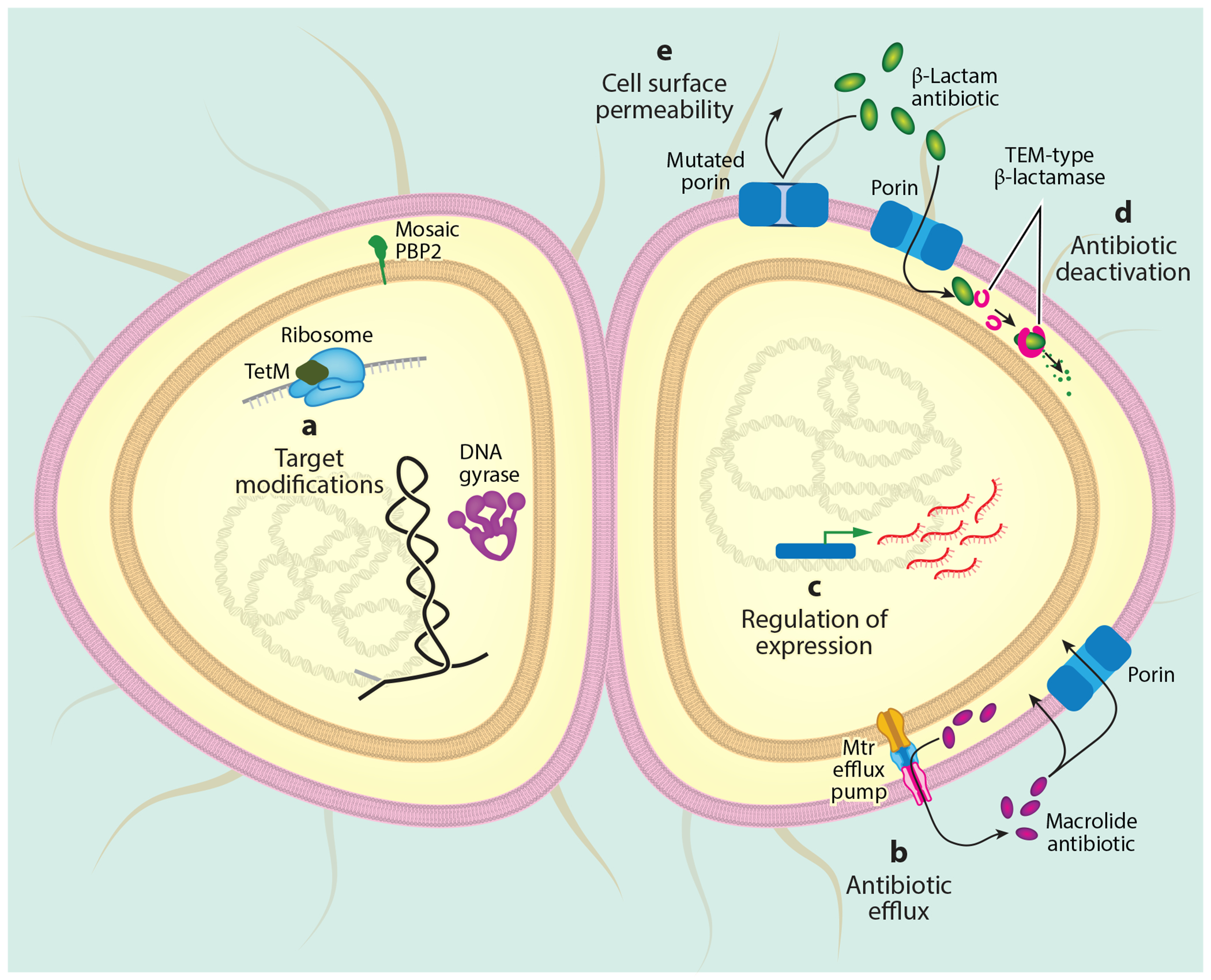
*Neisseria* have transferred multiple mutations encoding resistance across species’ boundaries, including mutations affecting (*a*) the drug target’s structure. For example, mutations in the gene encoding penicillin-binding protein 2 (PBP2), the gene encoding DNA gyrase subunit A, and several loci encoding ribosomal proteins disrupt or inhibit drug binding and impart reduced susceptibility to β-lactams, fluoroquinolones, and macrolides, respectively. The ribosome protection protein TetM (encoded by *tetM*) harbored on the plasmid pConj and passed across the *Neisseria* imparts tetracycline resistance by binding to the ribosome and displacing tetracycline class antibiotics. (*b*,*c*) Another transferred mechanism of resistance enhances drug efflux through mutations within the *mtrCDE* efflux pump operon—namely in the inner membrane component of the pump (encoded by *mtrD*); these mutations likely enhance drug binding and export and are coupled with mutations in the promoter region of *mtrCDE* that increase expression of the pump. (*d*) Finally, the plasmid p*bla* harbors a TEM-type β-lactamase, which inactivates β-lactams. Other resistance mechanisms have been described in *Neisseria*, such as (*e*) reduced cell surface permeability through porin mutations and additional drug target modifications (e.g., to PBP1); however, whether they have been transferred across the *Neisseria* has not yet been demonstrated.

Allelic exchange through transformation of commensal DNA has been an important mechanism of β-lactam and macrolide resistance acquisition for the pathogenic *Neisseria*. The first observation of commensal alleles imparting resistance in *N. gonorrhoeae* was reported in the late 1980s and came from an investigation of a strain isolate from Boston in 1984 (137). The strain displayed decreased susceptibility to penicillin and had a sequence with low similarity to native gonococcal alleles in the carboxy-terminal region of the transpeptidase domain of penicillin-binding protein 2 (PBP2, encoded by *penA*). PBP2 is one of four native gonococcal PBPs. The others include PBP1 (transpeptidase/transglycosylase, encoded by *ponA*), PBP3 (peptidoglycan-degrading enzyme, encoded by *dacB*), and PBP4 (carboxypeptidase/endopeptidase, encoded by *pbpG*) (139, 140). PBP2 (and homologs in other bacteria) is an important transpeptidase: It is required in the biosynthesis of peptidoglycan and is a lethal target of β-lactam antimicrobials, which exert a bactericidal effect by binding and inhibiting PBPs (Figure 2). Mosaic *penA* alleles were acquired from *N. cinerea* and *Neisseria perflava* (now also known as *N. subflava*) or from *N. perflava* alone (138, 158). Since the initial discovery of mosaic alleles, approximately 40 haplotypes have been generally classified as alleles I–XXXVI based on substitutions at 82 amino acid positions (105), and additional alleles (i.e., *penA-60*) have been reported (102, 115). Mosaic alleles in gonococci contain 60–70 amino acid substitutions compared with native gonococcal wild-type sequences. Intragenus transfer of *penA* alleles was also reported in penicillin-resistant *N. meningitidis* and *N. lactamica* isolates (17, 138).

Mosaic *penA* alleles also decrease susceptibility to ESCs, including ceftriaxone, the last remaining option for first-line empirical antimicrobial monotherapy for gonorrhea. In the early 2000s, gonococci with reduced susceptibility to cephalosporins, particularly to cefixime, were reported in Japan (9, 101); these isolates carried the mosaic *penA-10* allele. However, much of the contemporary spread of ESC reduced susceptibility is seen in isolates with a different mosaic allele, *penA-34* (XXXIV). The *penA-34* allele likely originated in Japan in the 1990s after recombination of *penA-10* with a commensal *Neisseria* species (likely *N. perflava*, *Neisseria sicca*, and/or *N. cinerea*) and then secondary recombination with wild-type *penA-1* (169). This allele is now prevalent globally (148), with gonococci carrying *penA-34* often expressing elevated MICs to ceftriaxone (≥0.125 μg/mL) and cefixime (≥0.25 μg/mL) (62). Key mutations in mosaic alleles that are important for intermediate-level reduced susceptibility to ESCs include I312M, V316T, N512Y, and G545S; additional substitutions contributing to high-level ceftriaxone resistance (MICs ≥ 0.5 μg/mL) and cefixime (MICs ≥ 1 μg/mL) were reported in F89 strains (WHO-Y; *penA-44*: A501P), H041 strains (WHO-X; *penA-41*: A311V, A316P, and T483S), and FC428 strain-derivatives (*penA-60*: A311V and T483S) (145, 149). The H041 isolate collected from Japan in 2009 has multiple contributing mutations in its mosaic *penA* gene and one of the highest reported MICs to ceftriaxone at 2–4 μg/mL. Concerningly, population prevalence of mosaic *penA* alleles and thus ESC reduced susceptibility is increasing in some countries. For example, in South Korea, mosaic *penA* prevalence increased from 1.1% to 23.9% from 2012 through 2017, and in Vietnam, it increased from 0% in 2011 to 27% in 2015 to 2016. Although ESC reduced susceptibility is still uncommon in other parts of the world (e.g., < 0.5% in 2020 in the United States), further dissemination of mosaic alleles or the introduction of new *penA* alleles still evolving in commensal *Neisseria* populations could limit the effectiveness of ceftriaxone as a treatment for gonococcal infections (158).

### Multiple-Transferable Resistance

Intragenus allelic exchange has also played an important role in the spread of resistance to azithromycin in contemporary gonococcal populations. Azithromycin binds the large subunit of the ribosome (50S) and prevents the translocation of the peptidyl-tRNA. Azithromycin, in conjunction with ceftriaxone, was recommended in 2012 as part of an effective dual-antimicrobial therapy for uncomplicated gonorrhea (168). However, this recommendation changed in 2018, when azithromycin reduced susceptibility passed the established 5% prevalence threshold (26). While the majority of resistance historically could be explained by mutations in the 23S rRNA azithromycin-binding sites (C2611T and A2059G), novel mosaic *mtr* sequences from commensals are now understood to play a large role in contemporary resistance spread (30, 62, 104) and can provide clinical resistance to azithromycin by themselves. The Mtr (multiple-transferable resistance) efflux pump is composed of the MtrC-MtrD-MtrE cell envelope proteins, which together export diverse hydrophobic antimicrobial agents such as antibiotics, nonionic detergents, antibacterial peptides, bile salts, and gonadal steroid hormones from the cell (see 75); overexpression of *mtrCDE* has been linked to resistance to diverse antimicrobial agents through increased drug export from the cell (11, 65). Mosaic alleles were first truly recognized in 2012, when a novel *mtrR* promoter sequence was described in a cluster of gonococcal isolates from Australia (*n* = 10 of 397 total) with reduced susceptibility to azithromycin (MIC ≥ 2 μg/mL) (150). However, mosaic alleles originated earlier: For example, mosaic *mtr* alleles were associated with an outbreak of azithromycin resistance in Kansas City, Missouri, from 1999 to 2000, though they were not named mosaics (71, 96). More recently, multiple mosaic *mtr* alleles were described, and they differed in the species they were acquired from (*N. lactamica, N. polysaccharea, N. cinerea*, and *N. meningitidis*) and the pump components recombined (120, 129, 163). The specific mechanism of resistance seems to be composed of two separate but interacting components: (*a*) changes to the *mtr* promoter that overexpress the components of the MtrCDE pump and (*b*) a MtrD E823K substitution that likely enhances antimicrobial binding (129, 163) (Figure 2).

The persistence and expansion of *mtr* mosaic alleles within gonococcal populations has been documented globally. For example, in the United States, the prevalence of mosaic *mtr* alleles increased after the recommendation to add azithromycin as part of the treatment for gonorrhea in 2012—from 1% before (2000 to 2011) to 6% after (2012 to 2013) the addition of the drug (62). By 2019, 21% of US gonococcal isolates harbored mosaic alleles and showed reduced azithromycin susceptibility. Concerningly, a recent study in New South Wales, Australia, estimated that 76% of the observed reduced susceptibility to azithromycin was derived from inheritance of mosaic *mtr* alleles (166). Furthermore, these alleles have been identified worldwide: A partial list of countries with circulating mosaics includes the United States (51, 62), Australia (166), Russia (74), South Africa (72), Argentina (57), Canada (37), the Netherlands (35), and Germany (12). The identification of *mtr* sequences from multiple other *Neisseria* species suggests that several introduction events into the gonococcal population occurred, and the persistence and expansion of these alleles globally suggest that little to no fitness cost is associated with harboring these mosaic alleles (49).

### Other Antibiotic Resistance Pathways

These examples of commensal *Neisseria* transferring clinically relevant resistance to *N. gonorrhoeae* emphasize the need to expand surveillance to commensals, as a complement to the continued surveillance of gonococci, both to assess the prevalence of resistance in the *Neisseria* genus and to determine the probability of novel resistance emergence that may affect future antibiotic development. For example, zoliflodacin is an antibiotic that inhibits DNA replication by binding and inhibiting DNA gyrase subunit B and that was evaluated in recently completed phase 3 clinical trials (58). Studies associated with the clinical trial and prior lab-based experiments suggested that *N. gonorrhoeae* had a low probability of developing decreased susceptibility to zoliflodacin (8, 50). However, Abdellati and colleagues (5) recently found that several commensal species (*N. mucosa, N. subflava*, and *N. cinerea* but not *N. lactamica*) developed point mutations in the *gyrB* gene that lead to decreased susceptibility (8- to 64-fold decrease) to zoliflodacin in vitro. Additionally, transformation of *N. gonorrhoeae* with DNA from evolved zoliflodacin-resistant *N. mucosa* strains decreased *N. gonorrhoeae*’s susceptibility by 4-fold through inheritance of altered *gyrB* haplotypes; this decrease suggests that these resistance alleles can promote zoliflodacin resistance in natural populations of gonococci. Indeed, in a recent global phylogenomic analysis of 20,000 gonococcal strains, extensive evidence supported recombination between *N. gonorrhoeae* and *Neisseria* commensals (130) in not only *gyrB* but also other loci affecting fluoroquinolone resistance (i.e., *gyrA* and *parC*/*parE*). Thus, we should profile commensals for resistance associated with not only current antimicrobial therapies but also those under development (92).

## RAPID SHUTTLES FOR GENETIC EXCHANGE: PLASMIDS AS ALTERNATIVE DRIVERS OF RESISTANCE ACQUISITION

Plasmids are small, circular, double-stranded DNA molecules that facilitate the transfer of genetic material from donor to recipient cells and allow the rapid spread of novel genotypes across both closely and distantly related bacterial lineages. These mobile genetic elements are spread through conjugation, a conserved mechanism of HGT across bacteria, which involves (*a*) expression of transfer (*tra*) genes for the formation of the type IV conjugative pilus and type IV secretion system, (*b*) processing of the plasmid (DNA nicking and linearization), (*c*) extrusion of single-stranded DNA through the conjugative pore into the recipient cell, and (*d*) plasmid recirculation and maintenance (Figure 1c) (23, 162). This process is contact dependent and thus typically occurs among bacteria inhabiting similar ecological niches. Within gonococci, there are three main classes of plasmids: a conjugative plasmid (pConj, as introduced above), a β-lactamase-containing plasmid (p*bla*), and a cryptic plasmid (pCryp) of unknown function (25, 98). The main contributors to high-level tetracycline and penicillin resistance are pConj and p*bla*, respectively, and they often cotransfer; their evolutionary origins highlight the importance of interspecific and intergenus plasmid exchange in *Neisseria* resistance acquisition.

pConj, a 39- to 43-kb plasmid, enables conjugation and has prevented the continued use of tetracycline and doxycycline for the treatment of gonorrhea (111). Conjugative plasmids are found in about 33% of gonococci in the United States and up to 90% of gonococcal isolates in some low- and middle-income countries (LMICs) because of widespread use of tetracycline, and three main types include a 24.5-MDa plasmid (pLE2451, with three different variants: pConj.5, pConj.6, and pConj.7) and two 25.2-MDa plasmids (Dutch type, with two variants: pConj.3 and pConj.4; and American type, with two variants: pConj.1 and pConj.2); these plasmids contain the *tetM* gene, encoding a ribosome protection protein, which imparts high-level tetracycline and doxycycline resistance (Figure 2) (24, 98, 111, 126, 171). The *tetM* determinants on gonococcal pConj plasmids likely originated from a class II Tn916-like streptococcal transposon, which subsequently had the gene encoding the transposase deleted but maintained the functionality of the *tetM* region (144), and some researchers speculate that independent *tetM* introduction events occurred on Dutch-type and American-type plasmids (54). Concerningly, 25.2-MDa, *tetM*-containing plasmids can be readily transferred from *N. gonorrhoeae* to the commensal *Neisseria* and back again to *N. gonorrhoeae* in the laboratory (125), and these plasmids have also been identified in natural commensal populations (171); these findings suggest that there are multiple potential sources of resistance for tetracycline-susceptible pathogenic *Neisseria*. pConj variants have also been identified in *N. meningitidis* isolates (approximately 1 in 200), and given the significantly greater genetic diversity across *N. meningitidis* clades compared with those of the other *Neisseria*, this identification implies that pConj evolved long-term in meningococcus and subsequently transferred to other members of the genus (171). Importantly, predictive mathematical models suggest that doxycycline postexposure prophylaxis (doxy-PEP) will spread resistance, since we would expect the expansion of *tetM*-containing pConj across all *Neisseria*, and this expansion would mobilize other resistance-harboring plasmids (i.e., p*bla*) and threaten the treatment of gonorrhea with β-lactams (13, 79, 119).

Plasmid-mediated penicillin resistance in gonococci originates from a 3.2- to 9.3-kb β-lactamase plasmid (p*bla*, as introduced above), which harbors TEM-type β-lactamases and is present in approximately 50% of isolates (Figure 2) (24, 123). p*bla* was acquired by the gonococcus from a likely pharyngeal-residing *Haemophilus* species (20, 46). First characterized in 1976, β-lactamase-producing plasmids are the main cause of high-level (MIC > 2 μg/mL) penicillin resistance (39). There are seven main types—Asia (7.4 kb), Africa (5.6 kb), Toronto/Rio (5.2 kb), Nîmes (6.8 kb), New Zealand (9.3 kb), Johannesburg (4.8 kb), and Australia (3.2 kb)—and the Asia type is considered the ancestral variant (100, 112). Most gonococcal penicillinase-producing strains carry *bla*_TEM-1_ or *bla*_TEM-135_ genes, which encode β-lactamase. Concerningly, these genes are just one (*bla*_TEM-135_ requires A185T) or two (*bla*_TEM-1_ requires M182T and S268G) amino acid substitutions away from becoming extended-spectrum β-lactamases (ESBLs) (45, 100, 131). These altered enzymes would be able to hydrolyze all ESCs—a nightmare scenario potentially leading to untreatable gonorrhea infections. In addition to being documented in gonococcal reservoirs, p*bla* has been described in commensal *Neisseria*, with one cross-sectional study finding *bla*_TEM-1_ in 93.9% of clinical isolates in India (142). Additional studies have documented p*bla* in *N. meningitidis* isolates (40, 142), and in vitro experiments have demonstrated that p*bla* can be transferred via conjugation from the gonococcus to other *Neisseria*, between meningococcal strains, and from *N. cinerea* to *N. gonorrhoeae* (19, 56, 126).

Given the ability of commensal *Neisseria* to harbor both pConj and p*bla* and the demonstrated cross-species exchange of these mobile genetic elements, commensal *Neisseria* must be considered as a donor of tetracycline and penicillin resistance through plasmid exchange. Additionally, commensal *Neisseria* may serve as an intermediate for plasmids containing ESBL-encoding genes gained from other respiratory tract pathogens. ESBL-producing plasmids are found in approximately 30 human-associated bacterial species that coevolve with commensal *Neisseria* in the oropharynx and nasopharynx (172). Thus, as we proposed previously, we believe there is a danger of commensal *Neisseria* acquiring (in novel permissible strain backgrounds) and transferring ESBL-producing plasmids to *N. gonorrhoeae* after continued “rolls of the evolutionary dice” (reshuffling and changing *Neisseria* strain genomic backgrounds), though this effect has not occurred yet (61).

## A BUBBLING CAULDRON OF NOVEL GENOTYPES: WHAT ELSE DO WE KNOW (AND NOT KNOW) ABOUT RESISTANCE IN COMMENSAL SPECIES?

Commensal *Neisseria* have been relatively underexplored for the resistance mechanisms they harbor, despite their importance (61). In a recent systematic review, Vanbaelen and colleagues (161) found that only 15 of 295 published studies on *Neisseria* commensals contained sufficient data to link phenotypic resistance profiles (i.e., MIC data) to genotypic data collected via whole-genome sequencing. Important conclusions garnered from this review include: (*a*) there is no evidence of intrinsic commensal resistance to the antimicrobials assessed (penicillin, ceftriaxone, ciprofloxacin, and azithromycin; i.e., historical isolates are more susceptible to antimicrobials), (*b*) resistance is increasing in commensal populations over time, and (*c*) surveillance of the commensal *Neisseria* will aid in limiting the spread of AMR to the pathogenic *Neisseria* through the detection of resistance alleles that could potentially spread across species’ boundaries. Indeed, surveillance of commensals may in fact be more important than that of pathogenic *Neisseria* as the former’s higher prevalence (carried in 100% of the human population) increases the probability of unintentional antimicrobial selection through bystander selection. Furthermore, commensals appear to be more resilient to antibiotic perturbations than do gonococci, with their abundance remaining remarkably consistent after antibiotic exposure (34, 78, 86). These close relatives thus serve as a “bubbling cauldron” of new adaptive solutions for the pathogenic *Neisseria* that are less frequently exposed (0.01% to 10% carriage rates) to nontargeted antimicrobial therapies (42).

A study of Vietnamese men showed direct selection for reduced drug susceptibility in commensal *Neisseria* populations after antibiotic usage, as well as an association between recent antibiotic use (within 1 month) and increased MICs to cefixime, ceftriaxone, and cefpodoxime in commensals compared with those in a control population that had no antibiotic use between 1 and 6 months prior to the study (42). In recent surveillance studies, rates of resistance in natural commensal populations also were high. For example, in Bologna, Italy, 90% of commensal strains had reduced susceptibility to azithromycin, 58% to ciprofloxacin, and 11% to ceftriaxone and cefotaxime (55). In a study in Belgium, reduced susceptibility to azithromycin, ciprofloxacin, and ceftriaxone was higher in commensals than in pathogenic *Neisseria* (87). As commensal *Neisseria* are readily shared among individuals with close contact (i.e., sexual partners or family members), resistant strains can be mobilized within contact networks (3, 160). Though these and other studies are beginning to shed light on the prevalence of resistance in commensal *Neisseria*, resistance profiling in commensals is in its infancy compared with that of pathogens (61, 160), and this situation emphasizes the need to implement surveillance and collection of commensal *Neisseria* through currently established surveillance systems for *N. gonorrhoeae*.

Aside from the mosaic *mtr* and *penA* alleles and the resistance carried on pConj and p*bla* plasmids, the particular mutations encoding resistance in commensals are not yet fully described (61, 161). One mutation characterized in natural commensal populations includes a T91I substitution in DNA gyrase subunit A (encoded by *gyrA*) and has been associated with elevated commensal ciprofloxacin MICs in vitro (69). This mutation has been described in *N. lactamica, N. cinerea*, and *N. subflava*, and recent HGT from these commensal species has played a major role in the global spread of ciprofloxacin resistance in meningococcal populations (Figure 2) (60, 76, 151). First reported in the United States in 2007, mosaic *gyrA* and *parC* (encoding topoisomerase IV) haplotypes in meningococci appear to have spread, with more than 53.4% of contemporary US quinolone-resistant meningococci (QRM) harboring mosaic alleles. Concerningly, the prevalence of QRM with mosaic *gyrA* and *parC* haplotypes exceeded 70% (28) in China, and 99% of 293 commensal *Neisseria* sampled were quinolone resistant; these findings suggest that quinolone resistance may have an especially high likelihood of interspecies transfer from commensal reservoirs to pathogens. Large population genomic surveys have also revealed a high propensity for HGT across the *Neisseria* of quinolone resistance–conferring loci (i.e., *gyrA*, *gyrB*, *parC*, and *parE*) (92).

Ribosomal protein–encoding loci also are a hotspot for interspecies allelic exchange. For example, in an analysis of 11,659 *Neisseria* genomes, the genes *rplB*, *rplD*, and *rplY*, which encode the ribosomal proteins L2, L4, and L25, respectively, showed evidence of cross-species recombination, with gonococcal and *N. cinerea* recipient strains inheriting *N. subflava* and *N. lactamica* sequences (93). Mosaic alleles were associated with reduced susceptibility to macrolides; interestingly, however, they first appeared in isolates from the 1930s, and thus their emergence cannot be linked to antibiotic use and suggests frequent recombination in these regions, even in the absence of a known selective pressure. In vitro evolution experiments also point to commensal ribosomal proteins as important contributors to reduced susceptibility to macrolides, with tandem duplications emerging in *rplD* (L4), in *rplV* (L22), and in the *rpmH* (L34) promoter region in multiple commensal species after macrolide selection (51, 118). Finally, MsrD, which displaces macrolides from the ribosome, was found in 9 of 11 *N. subflava* isolates from Belgium and was inherited from *Streptococcus pneumoniae* (34).

## THE DANGERS OF EXTERNAL PRESSURES ON COMMENSAL AND PATHOGENIC *NEISSERIA* EVOLUTION

The intended use of doxy-PEP, which involves the provision of doxycycline within 24–72 h of unprotected sex, is to reduce the risk of contracting and spreading the most common sexually transmitted infections (STIs; i.e., gonorrhea, chlamydia, and syphilis). Doxycycline is a broad-spectrum tetracycline antibiotic that inhibits protein production by binding allosterically to the 30S ribosomal subunit of bacterial ribosomes; however, because of the high carriage rate of *tetM*-containing pConj plasmids in the gonococcal population [e.g., approximately 33% in the United States and up to 90% in LMICs (24)], doxycycline is not recommended to treat gonococcal infections. Though doxy-PEP was trialed with some success in 2014–2016 and 2020–2022, members of the *Neisseria* research community are concerned that long-term use will select for multidrug-resistant gonococci (MDR-Ngo). In one US study, doxy-PEP implementation decreased the incidence of a diagnosis of at least one STI in quarterly medical visits by approximately 20% compared with standard care (90). Doxy-PEP also reduced the incidence of gonococcal infections by 10%; however, higher incidences of tetracycline-resistant *N. gonorrhoeae* were also reported in doxy-PEP participants compared with controls. In a study in France (IPERGAY), doxy-PEP reduced the incidence of most bacterial STIs in populations of high-risk men who have sex with men; however, it did not reduce the risk of contracting gonococcal infections (97), likely because 92% of gonococci in France are resistant to tetracycline (154). In recent mathematical models based on clinical trials, doxy-PEP decreased the incidence of gonococcal infections in the short term by limiting transmission of susceptible lineages but increased long-term infection prevalence as doxycycline-resistant lineages spread. These models predict the loss of doxy-PEP’s clinical efficacy for the treatment of gonorrhea in 10–20 years and also predict minimal impact on extending the usefulness of ceftriaxone as a treatment for gonorrhea (83, 119). Hence, we can expect that already prevalent gonococcal lineages harboring *tetM*-containing pConj plasmids will spread with doxycycline selection, render tetracycline class antibiotics even more ineffective, and perhaps more concerningly, may also promote the evolution of MDR-Ngo (99). Indeed, Ngo resistance will likely increase through doxy-PEP selection, as pConj contains all the necessary machinery for conjugation. Thus, we anticipate a higher likelihood of β-lactam resistance via HGT of p*bla* across gonococcal lineages; this resistance could be important for future treatment with ESCs, as some variants are one to two mutations away from ESBLs, and perhaps for unanticipated cross-species spread.

Doxy-PEP may have unintended consequences within the commensal *Neisseria* through bystander selection. Given that commensal *Neisseria* have a higher carriage rate (100%) compared with that of the pathogenic *Neisseria* (0.01% to 10%) (42, 79, 161), their exposure to unintentional antibiotic selection is higher. A single study has investigated the impact of doxycycline selection on *Neisseria* commensals, and this study found no evidence of resistance emergence in a 7-day span (for *N. gonorrhoeae* or *N. subflava*). However, *Klebsiella pneumoniae* and *Escherichia coli* displayed a 50-time increase in doxycycline resistance in the study, in addition to a 10-time increase in resistance to ceftriaxone and ciprofloxacin for *K. pneumoniae*. More recently, in vitro selection was shown to select for doxycycline resistance across four *Neisseria* commensals (127). We know that the host ranges of both the pConj and p*bla* plasmids extend across species’ boundaries, with transfer occurring between *Neisseria* species in vitro (125, 126) and their presence in different species also noted in natural populations (171). Thus, we expect that doxy-PEP will select for increased prevalence of pConj (and tetracycline resistance) and the often cotransferred p*bla* (and β-lactam resistance) in multiple *Neisseria* populations, along with uncharacterized chromosomal mutations. Indeed, doxycycline use recently was shown to select for resistance and pConj carriage in natural commensal populations (122). Therefore, we must be prepared for an expanded pool of doxycycline-selected resistance (both chromosomal and plasmid based) and MDR, from which susceptible gonococci can readily source resistance. As doxy-PEP is likely here to stay because of its effectiveness against *Chlamydia trachomatis* and *Treponema pallidum*, which cause chlamydia and syphilis infections, respectively (87, 95), alternative strategies must be considered to curtail the spread of AMR in *Neisseria* populations. In the absence of other approved antibiotics that would be effective against all three bacterial STI pathogens without causing the same concern about exacerbating resistance in *N. gonorrhoeae*, a multifaceted approach including both antibiotic-based and antibiotic-independent methods must be considered. In the short term, targeted vaccination of high-risk populations with meningococcal vaccines could reduce the incidence of gonorrhea in this cohort. Meningococcal vaccines targeting outer-membrane vesicles (OMVs) (i.e., MenB-4C) show some cross-protection against *N. gonorrhoeae* and 30–59% reductions in the incidence of gonococcal infections (2, 164). Currently, the impact of meningococcal vaccines on commensal populations is unclear, yet reducing gonococcal disease burden may slow the dissemination of doxy-PEP-selected resistance genotypes into pathogen populations. Complementary to this approach, continued and strategic public health education of high-risk populations about bacterial STIs (i.e., non-HIV) and STI prevention (i.e., condom use, meningococcal vaccines, etc.) will be critical to curtailing spread. In the long term, a dual approach must support the discovery and development of (*a*) new antibiotics targeting all three bacterial STI pathogens and (*b*) gonococcal vaccine research.

An additional contemporary pressure that will soon shape *Neisseria* evolution is the overprescription of azithromycin during the COVID-19 pandemic. Early in the pandemic, antibiotic use increased in US hospitals, often without evidence of an underlying bacterial infection, and about 80% of hospitalized patients with COVID-19 received at least one antibiotic, typically azithromycin and/or ceftriaxone (27). In outpatient prescriptions filled by Medicare holders, federally supported by the US government, from April 2020 to April 2021, azithromycin was by far the most frequently prescribed antibiotic (50.7%) (152). In nursing homes, azithromycin use was 1.5 times higher in April 2020 than in 2019 (27). The impact of the pandemic thus undermined years of careful antimicrobial stewardship, with downstream effects yet to be fully untangled, though some patterns appear to be emerging. Interestingly, a study in the Netherlands found an increase in the prevalence of low-level-resistant *N. gonorrhoeae* strains with mosaic *mtr* alleles since the pandemic (173). Genetic diversity has also been reduced in gonococcal populations, with fewer opportunities for transmission during lockdown (7). This world-pausing event undoubtedly had impacts on the dynamics of commensal communities as well, and we wonder if a higher prevalence of reduced susceptibility to azithromycin has also emerged in these populations.

An additional consideration in the near future will be the potential for resistance emergence to the novel antibiotic zoliflodacin in commensal populations. Though limited resistance emergence has been found in gonococci, in vitro evolution suggests that commensals readily evolve resistance through *gyrB* mutations (i.e., G28C, M29I, D429N, K450I, S467N, M464R, and T472P), some of which are transferable to *N. gonorrhoeae* strains (5). Even if these mutations cause fitness defects in gonococci now, compensatory mutations seem likely to occur and to permit more permissive exchange across species. Finally, we wonder how antigonococcal OMV-based vaccines will affect adaptive evolution of our resident *Neisseria* in the future, given cross-reactivity and the wide use of treatments for other pandemic-level diseases [e.g, type 2 diabetes (133)] through altered host physiology. Thus, as Kenyon and colleagues (78) proposed, taking a “pan-*Neisseria*” approach will stand the best chance of selecting the most effective current and future antibiotics for the treatment of gonococcal infection.

## AM I REALLY THAT BAD? COMMENSALS AS TOOLS TO COMBAT ANTIMICROBIAL RESISTANCE IN GONOCOCCI

While commensal *Neisseria* pose a newly recognized threat to AMR acquisition in pathogenic *Neisseria*, they also serve as an underexplored reservoir of potential solutions to this very same danger. For example, can we utilize the “tribal warfare” and DNA-based killing of gonococci by commensal *Neisseria* (135) and/or other competitive mechanisms as a potential probiotic therapy that may limit colonization rates of pathogenic *Neisseria*? Could inoculation with specific species (perhaps even specific strains engineered to survive in the urogenital tract) provide gonococcal protection? Indeed, *N*. *cinerea* colocalizes with *N*. *meningitidis* on epithelial cells and has been shown to both limit the size of meningococcal microcolonies and impair effective colonization of host cells by *N*. *meningitidis* (33). Additionally, commensal *Neisseria* carriers have been shown to develop opsonophagocytic antibodies protective against *N. meningitidis* (47). Finally, *N. mucosa* has been suggested to produce secondary metabolites inhibitory to gonococcal growth (6), though this last report was recently contested (4). Therefore, a commensal *Neisseria* probiotic may reduce carriage rates of gonococci if it could be delivered to the urogenital tract (UGT) niche. Engineering a UGT commensal strain may be feasible given the recent natural acquisition of the gonococcal *aniA/norB* cassette by serogroup C *N. meningitidis* strains, with this acquisition leading to the lineages’ rapid adaptation to anaerobic conditions, the expansion of these lineages in the urogenital niche, and the provision of a model that we may be able to follow (122, 153).

Furthermore, commensal *Neisseria* are likely unexplored resources for other important antigonococcal tools such as prophages, growth-inhibiting properties including secondary metabolites, and potential vaccine targets. Notably, intact unique prophage sequences have been identified in commensal *Neisseria* genomes (107). Additionally, exploitation of the “tribal warfare” between *Neisseria* could also be a key tool in inhibiting the growth of pathogenic *Neisseria*, through either DNA-dependent killing (e.g., through the integration of differentially methylated DNA into sexual lubricants) (121) or the production of other antigonococcal antibiotics. Finally, do commensals secrete OMVs, and could these be used as vaccine deliverables in the pharyngeal niche, which has low antibiotic bioavailability?

## CONCLUSION

In this article, we highlight the importance of commensal *Neisseria* in the spread and establishment of AMR mechanisms in the sexually transmitted pathogen *N. gonorrhoeae*. We summarize current findings on AMR mechanisms identified in *N. gonorrhoeae* and commensal *Neisseria* species, describe the possible mechanisms that allow *Neisseria* species to exchange these genes, and discuss doxycycline as prophylaxis. We posit that the expansion of our surveillance efforts on *N. gonorrhoeae* to include commensal *Neisseria* species is important. Through the surveillance and collection of commensals, we would improve our understanding of host–commensal, pathogen–commensal (for a comprehensive review of interspecies interactions, see 36), and host–pathogen interactions; provide new avenues of prevention and treatment strategies; and assess the breadth of AMR in commensal species; this work could help predict future AMR acquisition in the pathogenic species.

## Figures and Tables

**Table 1:** Reported mutations contributing to reduced antibiotic susceptibility in gonococci and the commensal *Neisseria*

Antibiotic	Locus or loci	Mutation(s) in Ngo^a^	Mutation(s) in cN	Reference(s)
Azithromycin	*mtrR*	A39T, G45D	A37V^b^, G172D^b^	65
	*mtrRCDE* promoter	A56C, ΔA57, G131A, G120A, (*mtr120*)	NR	65, 163
	*mtrD*	mosaic alleles, E823K	E823K	129, 163
	*ermA, ermB, ermC, ermF* genes	+	+	124
	23S rRNA	(C2611T), (A2059G)	NR	30, 104
	*macAB* promoter	(G48T)	NR	157
	*rplV*	91-KRFQARAKG, 91-GRGNRIQARAKG	78-KGPSLKRFQARA^b^	51, 62
	*rplD*	G68D/C, G70D, others	NR	91
	*rpmH*	7-PSVTKRKR, 7-PSVTNTYQP, 7-LKRTYQ	6-HHETHLS, 6-QLMKRTYQ, 8-SVKRTYQP	51, 86, 118
	*rpsC*	NR	(TD promoter)	118
Cefixime	*penA*	mosaic alleles, A501V/T	A311V, I312M, V316P/T, P399S^b^, A501T, F504L, A510V, A516G, V574E^b^, A581S^b^, F518S^b^, G542S, V548E^b^, A549E^b^, P551L	9, 49
Ceftriaxone	*penA*	mosaic alleles, A501V/T	A311V, I312M, V316P/T, P399S^b^, A501T, F504L, A510V, A516G, V574E^b^, A581S^b^, F518S^b^, G542S, V548E^b^, A549E^b^, P551L	9, 49
	*mtrR*	G45D, (A-deletion in the promoter)	NR	172
	*rpoB*	R201H	NR	113
	*rpoD*	E98K, Δ92–95	NR	113
Ciprofloxacin	*gyrA*	S91F, D95A/N/G	G89D^b^, S91N/R^b^, T91I	62, 127
	*gyrB*	D429N	P739H^b^	127, 130
	*parC*	D86N, S87R/N, E91K	G85C^b^	127, 157
	*parE*	G410V	R320S^b^	127, 157
	*mtrR*	A39T, G45D, Y105H	T11N^b^, T93P^b^, G45S^b^	127
	*norM*	(−35 promoter sequence)	NR	157
Gentamicin	*fusA*	A563V, G564D, V651F	NR	67
Penicillin	*penA*	mosaic alleles	A311V, I312M, V316P/T, P399S^b^, A501T, F504L, A510V, A516G, V574E^b^, A581S^b^, F518S^b^, G542S, V548E^b^, A549E^b^, P551L	51, 149
	*porB*	G120K, A121D/N	G120K/D, A121D	49, 157
	*mtrR*	A39T, G45D	T11I^b^, A39T, G45D	49, 51, 65
	*mtrRCDE* promoter	(ΔA56C), (ΔA57), (G131A), (G120A), (*mtr120*)	NR	157
	*mtrD*	NR	V139G^b^, F604I^b^, L996I^b^, A1009T^b^	51
	*^bla^*TEM	+	+	13, 45, 171
	*ponA*	L421P	NR	157
	*pilQ*	E666K	NR	103
	*rpoB*	NR	E345A^b^, P591S^b^	51
Spectinomycin	*rpsE*	T24P, ΔV25, K26E	NR	157
Sulfonamides	*folP*	R228	V48A, P64L, P64S	49, 157
Tetracycline/doxycycline	*porB*	G120K, A121D/N	G120K/D, A121D	49
	*mtrR*	G45D	G45D, A46T^b^	49, 51, 65
	*tetM*	+	+	98, 103, 171
	*pilQ*	E666K	NR	103
	*rpsJ*	V57M	V57M^b^	65, 127
Zoliflodacin	*gyrB*	D429V, S467N, K450	NR	130

a Protein mutations. Entries within parentheses are DNA mutations.

b Mutation derived via in vitro selection and not yet validated as causal or as present in natural populations.

A plus sign indicates the presence of a locus.

Abbreviations: cN, commensal *Neisseria*; Δ, deletion; Ngo, gonococci; NR, not reported; TD, tandem duplications.
